# Napthyridine-derived compounds as promising inhibitors for *Staphylococcus aureus* CrtM: a primer for the discovery of potential anti-*Staphylococcus aureus* agents

**DOI:** 10.3389/fmicb.2023.1279082

**Published:** 2023-10-24

**Authors:** Mohammed Bourhia, Muhammad Shahab, Guojun Zheng, Yousef A. Bin Jardan, Baye Sitotaw, Lahcen Ouahmane, Farid Khallouki

**Affiliations:** ^1^Department of Chemistry and Biochemistry, Faculty of Medicine and Pharmacy, Ibn Zohr University, Laayoune, Morocco; ^2^State Key Laboratories of Chemical Resources Engineering, Beijing University of Chemical Technology, Beijing, China; ^3^Department of Pharmaceutics, College of Pharmacy, King Saud University, Riyadh, Saudi Arabia; ^4^Department of Biology, Bahir Dar University, Bahir Dar, Ethiopia; ^5^Laboratory of Microbial Biotechnologies, Agrosciences and Environment (BioMAgE), Labeled Research Unit-CNRSTN°4, Cadi Ayyad University, Marrakesh, Morocco; ^6^Department of Biology, FSTE, University Moulay Ismail, Errachidia, Morocco

**Keywords:** *Staphylococcus aureus*, napthyridine, molecular docking, molecular dynamics simulation, quantum mechanical calculation

## Abstract

The disease-free existence of humans is constantly under attack by a variety of infections caused by a variety of organisms including bacteria. Notable among the bacteria is *Staphylococcus aureus* which is an etiological organism for infections including impetigo, folliculitis, and furuncles. The response of the human immune system against this disease is often neutralized by the production of a pigment called Staphyloxanthin (STX) via a series of reactions mediated by several enzymes. Among these enzymes, dehydrosqualene synthase, also known as CrtM, has emerged as a viable drug target due to its role in mediating the first step of the pathway. Consequently, this study employs molecular modeling approaches including molecular docking, quantum mechanical calculations, and molecular dynamics (MD) simulations among others to investigate the potential of napthyridine derivatives to serve as inhibitors of the CrtM. The results of the study revealed the high binding affinities of the compounds for the target as demonstrated by their docking scores, while further subjection to screening pipeline aimed at determining their fitness for development into drugs revealed just one compound namely 6-[[1-[(2-fluorophenyl) methyl]triazol-4-yl]methoxy]-4-oxo-1H-1,5-naphthyridine-3-carboxylic acid as the compound with good drug-like, pharmacokinetics, and toxicity properties profiles. A 100 ns-long MD simulation of the complexes formed after molecular docking revealed the stable interaction of the compound with the target. Ultimately, this study can be a promising outlet to discover a weapon to fight against clinically resistant bacteria, however, further experimental studies are suggested to carry out in the wet lab, pre-clinical, and clinical levels.

## 1. Introduction

Of all the limited number of bacteria that cause diseases in humans, *Staphylococcus aureus* has risen to prominence due to its multi-pathogenic ability among many other reasons. Notably, *S. aureus* is a gram-positive bacterium that can cause a wide range of infections in humans. It is a significant pathogen, both in community settings and healthcare facilities, leading to morbidity and mortality in affected individuals (Tong et al., 2015). *Staphylococcus aureus* can be transmitted through direct contact with infected individuals or carriers. Additionally, it can spread via contaminated objects or surfaces, leading to person-to-person transmission in households and communities (Knox et al., 2015). In healthcare settings, the bacterium can spread among patients, healthcare workers, and visitors, contributing to hospital-acquired infections (Solberg, 2000). *S. aureus* infections can manifest in diverse clinical presentations. Common diseases caused by this bacterium include skin and soft tissue infections, such as impetigo, folliculitis, and furuncles (Knox et al., 2015). In some cases, *S. aureus* infections can cause pneumonia, osteomyelitis, endocarditis, and sepsis (Knox et al., 2015). The bacterium is also associated with non-gonococcal urethritis, a condition characterized by inflammation of the urethra, particularly in regions like Nigeria, where *S. aureus* has been identified as a possible major cause of this condition (Oboho, 1984).

Upon human infection, the innate immune system of humans responds accordingly, triggering responses that often culminate in the production of reactive oxygen species (ROS) by neutrophils and macrophages. However, *S. aureus* reacts and disables the ROS via the production of a golden carotenoid pigment known as Staphyloxanthin (STX), hence, rendering the bacterium resistant to innate immune clearance (Liu et al., 2005; Clauditz et al., 2006). Studies have reported the pivotal role of STX in infectivity, as bacteria lacking the pigment were vulnerable to neutrophil killing and unable to produce disease in mouse skin and systemic infection models (Liu et al., 2008). Consequently, STX biosynthesis inhibition has emerged as a viable means for preventing and treating *S. aureus* infections. Notably, the first step in STX biosynthesis is mediated by the enzyme dehydrosqualene synthase, also known as diapophytoene synthase or CrtM. This process includes the head-to-head condensation of two molecules of farnesyl diphosphate (FPP) to produce the C30 species presqualene diphosphate, which is then transformed into dehydrosqualene (Zhang et al., 2018). The inhibition of CrtM has been demonstrated to increase the susceptibility of *S. aureus* to neutrophil killing and immune clearance, hence, rendering its inhibition a rational approach to therapeutics discovery (Liu et al., 2008).

The combination of the infections caused by *S. aureus* is done via the use of antimicrobials such as penicillin and vancomycin, however, the bacterium has evolved resistance mechanisms against these drugs, hence, limiting their efficacy and clinical usage (McCallum et al., 2010). Notably, the resistant strains of *S. aureus* include the multi-drug resistant strain (Renz and Dräger, 2021), the methicillin-resistant strains (Lee et al., 2018), and trimethoprim-resistant strains (Foster, 2017). The mechanisms of drug resistance utilized by this bacterium include the alteration of the target site of antibiotics, reducing intracellular concentration of antibiotics through the action of efflux pumps, overexpression of proteins with low affinities for drugs such as methicillin, and the acquisition of resistance genes (Pantosti et al., 2007; Peacock and Paterson, 2015; Foster, 2017; Yılmaz and Aslantaş, 2017). Consequently, newer classes of antimicrobials are still being searched against this bacterium. Napthyridine derivatives are a group of compounds that have garnered considerable attention due to their diverse and interesting biological activities. These derivatives demonstrate an array of pharmacological properties, including antimicrobial, antiviral, anticancer, anti-inflammatory, and analgesic activities, among many others (Madaan et al., 2015; Chukwuemeka et al., 2021; Olukunle et al., 2023). Exemplifying their antimicrobial property is a study in which they increased the susceptibility of multi-resistant bacterial strains to antibiotic treatment (Araújo-Neto et al., 2021). Hence, further exploration of their antimicrobial potential is still ongoing.

Historically, traditional drug discovery relied on empirical methods, including the use of traditional remedies and chance discoveries, such as aspirin derived from willow bark. Later, chemical libraries of synthetic and natural compounds were screened to identify potential therapeutic effects. However, this traditional approach had limitations, including increased costs due to the synthesis of numerous structural derivatives for each drug candidate (Gurung et al., 2021; Omoboyede et al., 2023a). The advent of computer-aided drug design (CADD) revolutionized drug discovery and development, introducing a new paradigm that utilizes computational techniques and algorithms to expedite the identification of potential drug candidates (Kumar, 2022; Omoboyede et al., 2023b). Using CADD techniques such as molecular docking, *in silico* pharmacokinetics properties evaluation, quantum mechanical (QM) calculations, and molecular dynamics simulation, this study aims to unravel newer classes of potential CrtM inhibitors that could be further explored in drug discovery odysseys.

## 2. Methods

### 2.1. Ligand identification and structure retrieval

Napthyridine derivatives were identified by searching the PubChem database^1^ with the keyword “Napthyridine.” The structures of the identified derivatives were then retrieved in structure data format (SDF) (Kim et al., 2019).

### 2.2. Target structure retrieval and preparation

The tertiary structure of CrtM was obtained by X-ray diffraction method and was retrieved from the Protein Databank (PDB)^2^ (Berman, 2000) in PDB format using the PDB ID: 2ZY1 (Song et al., 2009). The retrieved protein structure was subjected to preparatory procedures using the Dock prep module of the UCSF Chimera v1.10.2 software program (Pettersen et al., 2004). Notable procedures implemented during the preparatory steps included the deletion of heteroatoms, elimination of water molecules and the cognate ligand. Furthermore, charge ions and missing hydrogen atoms were added. Subsequently, the resulting structure was taken for energy minimization using an energy minimization algorithm of the Swiss-PdbViewer v.4.10 software program (Guex and Peitsch, 1997). The energy minimization was performed *in vacuo* with the GROMOS 43B1 parameter set and without field reaction. Noteworthy, the program automatically identifies missing side chains in protein residues and fixes them.

### 2.3. Molecular docking simulation

The affinity of the compounds and their interaction with the active site residues of CrtM was studied using molecular docking simulation using the VINA module in PyRx v 0.8 freeware (Trott and Olson, 2009). Prior to the molecular docking simulation, the active site residues of the protein were identified using the BIOVIA Discovery studio visualization, based on the space occupied by the co-crystalized ligand. Based on the position occupied by the identified active site residues, a grid box of size *x* = 18.6271077264 Å, *y* = 21.5963427287 Å, and *z* = 18.6762057027 Å, with a center dimension of *x* = 14.309490452, *y* = 54.634826077, and *z* = − 39.8837971487 were set to define the active site. An exhaustiveness of “8” was used during the docking simulation, and the complexes formed after docking were visualized using PyMOL v2.4.1 and LigPlot v2.2.4 for the 3D and 2D complexes, respectively (Wallace et al., 1995; DeLano, 2002). Ultimately, the docking protocol was validated by redocking the co-crystallized ligand against the protein and superimposing it on the undocked co-crystallized ligand to obtain the root mean square of deviation (RMSD).

### 2.4. Druglikeness and pharmacokinetics properties profiling

The druglikeness of the hit compounds obtained after molecular docking was assessed based on Lipinski’s rule of five (Ro5) (Lipinski et al., 1997/2001) using the SwissADME webserver (Daina et al., 2017).^3^ Subsequently, a comprehensive investigation of the absorption, distribution, metabolism, and excretion (ADME) properties of the drug-like compounds was conducted by employing the pkCSM webserver (Pires et al., 2015).^4^ Additionally, the toxicity potentials of the compounds were studied using the ProTox-II webserver (Banerjee et al., 2018).^5^

### 2.5. Quantum mechanical calculations

Quantum mechanical calculations were performed for the lead compounds identified in this study using the Molecular Orbital Package (MOPAC) software. Firstly, the structures of the compounds were subjected to protonation at pH 7.4, after which they were subjected to another pre-optimization procedure using the Merck Molecular Force Field 94 (MMF94) within the Avogadro v1.2.0 software program (Hanwell et al., 2012). Subsequently, the pre-optimized geometries of the compounds were used for the QM calculations using the PM7 semi-empirical Hamiltonian level (Dutra et al., 2013), with an implicit COSMO solvation model (Klamt and Schuurmann, 1993). Notably, geometric optimization of the structures at the semi-empirical theory level was accomplished through the utilization of the Broyden-Fletcher-Goldfarb-Shanno (BFGS) geometry optimizer. The Jmol software program (Hanson, 2010) was employed to visualize the charge distribution diagram of the frontier molecular orbitals (FMOs) and the molecular electrostatic potential (MEP) while various quantum chemical reactivity descriptors were calculated based on the energies of the highest occupied and lowest unoccupied molecular orbitals (E_HOMO_-_LUMO_).

### 2.6. Molecular dynamics simulation

The rigorous computer simulation method, Molecular Dynamics (MD) simulation, was employed to gain comprehensive insights into the stability of the complexes formed by the lead compound of this study and the control drug (Methicillin) with the target. The complexes were subjected to a rigorous 100 ns MD simulation using the Amber v2022 software with the ff14SB force field (Shahab et al., 2023). All the topology files and coordinate files were prepared using the tLEAP module. The Transferable intermolecular potential with 3 points (TIP3P) water model with a box dimension of 12.0 was used to adequately solvate each system. The counter-ions, such as sodium or chloride ions, were then delivered into the solvate box by the xleap in order to neutralize the systems. In addition, 1,000 steps of steepest descent minimization and 500 steps of conjugate gradient minimization were applied to the neutralized complexes Then, for the next 50 ps, each system was gradually heated to 300 K. A total of 100 ns of MD was run at constant pressure. The temperature was regulated by a Langevin thermostat (1 atm, 300 K) (Shahab et al., 2023). The Particle Mesh Ewald (PME) algorithm was used to calculate long-range interactions. For the covalent bonding, the SHAKE algorithm was employed (Shahab et al., 2023). Using the accelerated GPU pmemd, the whole MD for all the systems was completed. Cuda and the CPPTRAJ module of Amber 20 were used to evaluate the trajectories.

## 3. Results and discussion

### 3.1. Retrieved napthyridine derivatives

Sixty-five napthyridine derivatives were identified following a comprehensive search of the PubChem database and their structures were retrieved thereafter. Notably, these derivatives included 1, 5-Napthyridine, 1,6-Napthyridine, 1,7-Napthyridine, 1,7-Napthyridine, and 1,8-Napthyridine derivatives. Notably, the napthyridine derivatives are reputed for their array of pharmacological properties which include anticancer and much more important in this context, their antimicrobial properties, which included the sensitization of antimicrobial-resistant strains of *S. aureus* to existing drugs (Madaan et al., 2015; Araújo-Neto et al., 2021). The retrieved structures were prepared for molecular docking using the Open Babel incorporated into PyRx.

### 3.2. Retrieved target structure

**Figure 1:**
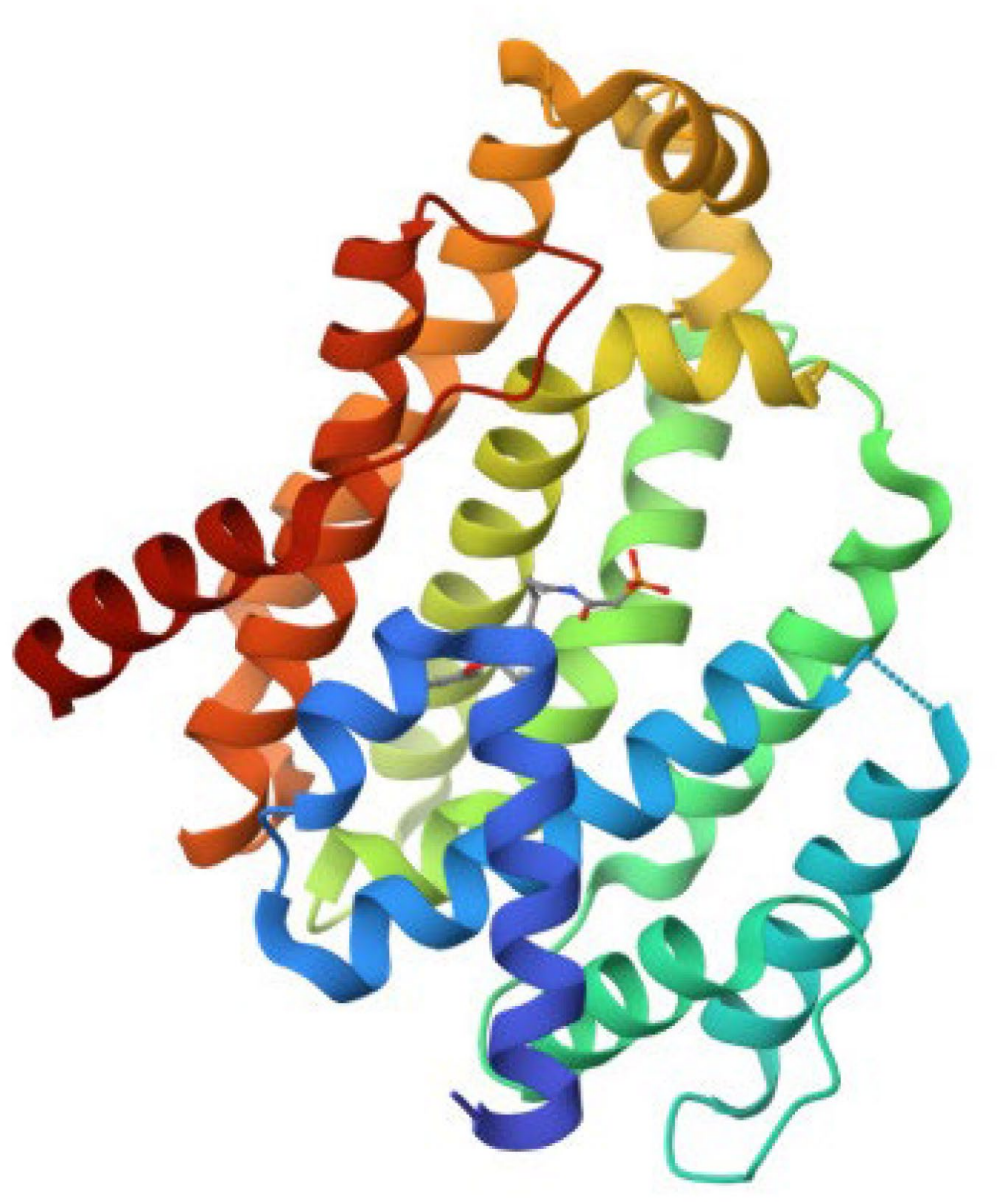
The retrieved three-dimensional structure of CrtM with the PDB ID 2ZY1.

The retrieved structure of the target from the PDB was elucidated using the X-ray diffraction method at an impressive resolution of 1.78 Å. The structure contained a single chain of 293 amino acid residues and was complexed with dipotassium (2-oxo-2-{[3-(3-phenoxyphenyl) propyl] aminoethyl) phosphonate. The retrieved structure was also subjected to a rigorous preparatory prior to molecular docking to ensure the protein was in the right conformational state. Figure 1 depicts the structure of the protein.

### 3.3. Molecular docking simulation

Molecular docking simulation was employed to study the interaction of the retrieved napthyridine derivatives with the active site amino acid residues of the target. As evident in Table 1, the docking scores of the compounds, which reveal their binding affinities for the target revealed many of the compounds were potent binders of the target. Compounds with the PubChem CID “136259988” and “44130717” were found to possess the highest affinity for the target with docking scores of −10.6 kcal/mol, while other compounds including “44130601” and “58628188” also had docking scores of −10.2 kcal/mol. Interestingly, the docking scores of the sixty-five compounds ranged from −4.1 kcal/mol to −10.6 kcal/mol, suggesting the compounds could potentially serve as potential inhibitors of the compounds. For subsequent downstream analyses, the compounds with the lowest five docking scores were selected. The selected exhibited docking scores ranging from −10.2 kcal/mol to −10.6 kcal/mol, while the standard drug (Methicillin) with the CID “6087”, employed as the control for the docking study had a docking score of −7.6 kcal/mol. It is worth noting that a lower docking score is indicative of a superior binding affinity.

**Table 1:** The docking scores of the napthyridine derivatives against CrtM including the standard drug (Methicillin).

Compounds CID	Docking scores (kcal/mol)
136259988	−10.6
44130717	−10.6
44130601	−10.3
58628188	−10.3
44130605	−10.2
44130716	−10.1
51001532	−10.1
22346280	−10
23284638	−10
44130606	−10
44130718	−10
20816879	−9.9
22346361	−9.9
22346421	−9.9
44130602	−9.9
59811153	−9.8
66997784	−9.8
14555496	−9.7
22346320	−9.7
44130719	−9.7
44130603	−9.6
13772748	−9.5
44130604	−9.5
10571743	−9.4
22346292	−9.4
58955169	−9.4
59472978	−9.4
2782953	−9.3
22346299	−9.2
22346301	−9.2
59811133	−9.1
59811134	−9.1
59811137	−9.1
10546084	−8.9
20296085	−8.8
57561127	−8.7
135121231	−8.3
13772732	−8.3
58926753	−8.3
15488572	−8.1
21883627	−8.1
23284635	−8
9965471	−8
12790859	−7.8
86587464	−7.8
11055142	−7.6
2777539	−7.6
6087	−7.6
2777542	−7.4
15189037	−7.3
10899616	−7.1
15592374	−7.1
85199	−7
89995619	−6.8
12204233	−6.7
2761050	−6.6
12018701	−6.5
22397149	−6.5
22503228	−6.5
12358230	−6.3
58387604	−6.3
12018700	−6.2
12018702	−6
136069	−6
10129935	−4.1

**Figure 2:**
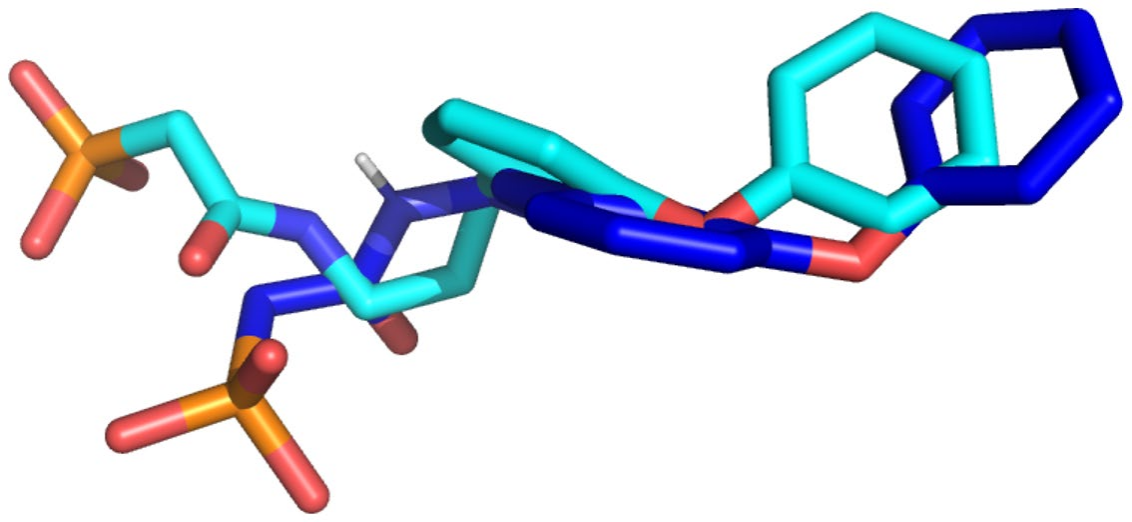
The superimposed X-ray crystallography pose and re-docked ligands. The X-ray crystallography and re-docked pose are colored cyan and blue, respectively.

The docking protocol was validated by the superimposition of the docked native ligand on the undocked native ligand, the RMSD value was calculated to be 0.056 Å, depicting the reliability of the docking protocol utilized in this study. Figure 2 depicts the superimposed docked and undocked native ligands.

### 3.4. Drug-likeness assessment

The hit compounds obtained from the molecular docking study were subjected to the Ro5 to evaluate their likelihood to serve as orally bioavailable drugs. This test takes into consideration the physicochemical properties of a compound including the molecular weight (MW), number of hydrogen bond donors (nHBD), number of hydrogen bond acceptors (nHBA), and octanol-to-water partition coefficient (MLogp), with compounds violating more than of the thresholds MW > 500 da, nHBD >5, nHBA >10, and MlogP >5 considered to be non-drug-like, and are potentially incapable of being absorbed into the bloodstream to reach their target site when taken orally. The results of this assessment are presented in Table 2. As evident in Table 2, all the compounds passed the Ro5 test, hence, rendering them fit as potential oral drugs. Notably, all the compounds passed with zero violations, except the compound with the CID “58628188”, which possessed a MW greater than 500 Da. These compounds were then subjected to further analyses.

**Table 2:** The druglikeness evaluation of the hit compounds and the standard drug (Clomipramine).

Phytochemical	MW (Da)	NHBA	NHBD	MLOGP	Violations
1-ethyl-N-(2-hydroxy-1-phenylindol-3-yl)imino-7-methyl-4-oxo-1,8-naphthyridine-3-carboxamide	451.48	6	1	3.34	0
6-[[1-[(2-fluorophenyl)methyl]triazol-4-yl]methoxy]-4-oxo-1H-1,5-naphthyridine-3-carboxylic acid	395.34	8	2	1.01	0
6-[(1-benzyltriazol-4-yl)methoxy]-4-oxo-1H-1,5-naphthyridine-3-carboxylic acid	377.35	7	2	0.89	0
ethyl 5-[4-(trifluoromethyl)anilino]-2-[3-(trifluoromethyl)pyridin-2-yl]-1,6-naphthyridine-7-carboxylate	506.40	11	1	3.65	1
6-[[1-[(4-methylphenyl)methyl]triazol-4-yl]methoxy]-4-oxo-1H-1,5-naphthyridine-3-carboxylic acid	391.38	7	2	1.12	0
Methicillin	380.42	6	2	1.01	0

### 3.5. Molecular interaction profiling

**Figure 3:**
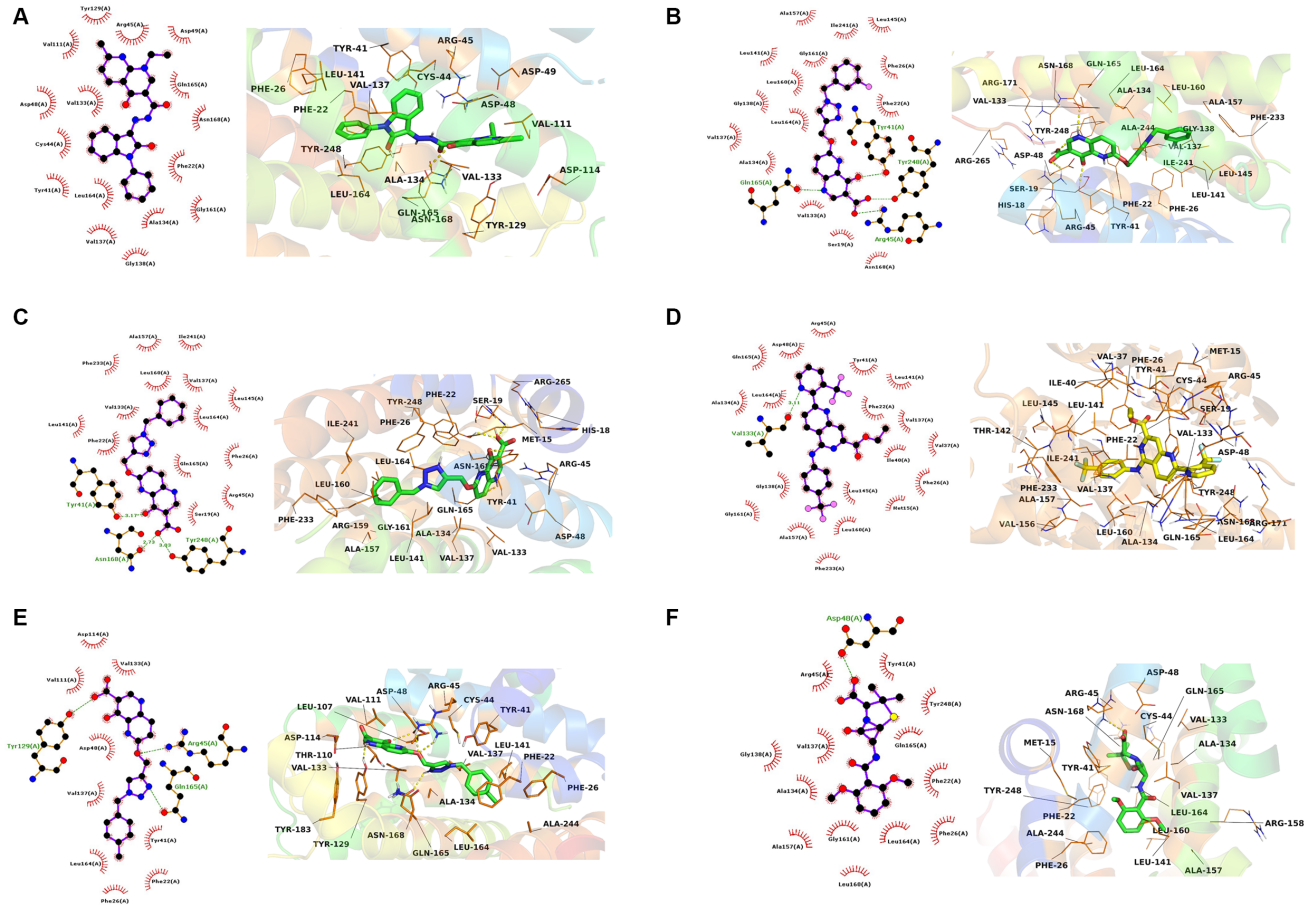
The 2D and 3D interaction profiles of the druglike hit compounds. **(A)** CrtM-136259988, **(B)** CrtM-44130717, **(C)** CrtM-44130601, **(D)** CrtM-58628188, **(E)** CrtM-44130716, **(F)** CrtM- Methicillin.

The interactions of the hit compounds with CrtM were visualized to get insights into the interactions compounds with the amino acid residues at the active site of the protein. As depicted in Figure 2, the complex’s interaction was major via hydrophobic interactions, however, hydrogen bonds were formed between some complexes. The compound with the CID “136259988” interacted with the active site amino acid residues of CrtM via hydrophobic bonds with Phe22, Tyr41, Cys44, Arg45, Asp48, Asp49, Val111, Tyr129, Val133, Ala134, Val137, Gly138, Gly161, Leu164, Gln165, and Asn168. Conversely, the compound with the CID “44130717” interacted with Tyr41, Asn168, and Tyr248 via hydrogen bonds while it interacted with Ser19, Phe22, Phe26, Arg45, Val133, Val137, Leu141, Leu145, Ala157, Leu160, Leu164, Gln165, Phe233, and Ile241 (Figure 3).

The compound with the CID “44130601” was found to exhibit the same interaction as that of CID “44130717,” but further interacted with Gly138 and Gly161. The compound with the CID “58628188” also maintained a similar interaction profile but interacted with Arg45 via hydrogen bonding as opposed to the hydrophobic interaction in the CrtM-44130601 complex, while also interacting with Ala134. The compound with the CID “44130716” and the standard drug (Methicillin) had very similar interactions. Specifically, both interacted with residues such as Phe22, Phe26, Tyr41, Val137, and Gly138 via hydrophobic interactions while the compound with the CID “44130716” interacted with Arg45, Tyr129 and Leu164 via hydrogen bonds, with Methicillin maintaining converse interactions with both Arg45 and Leu164. Also, Methicillin formed a hydrogen bond interaction with Asp48 and maintained hydrophobic interactions with Ala134, Gly138, Ala157, Leu160, Gly161, and Tyr248 while CID “44130716” further interacted with Val111, Asp114, Tyr129, and Val133 via hydrophobic interactions.

Interestingly, the interactions of the hit compounds of this study with the target were also found to be present in the interaction profile of hit compounds derived from other studies. Exemplifying this is the study of Hafidi et al. in which they reported that their hit compounds with residues including Asp48, Leu145, Ala157, Gln165, and Phe233, with the bulk of the interactions being hydrophobic (Hafidi et al., 2021). Similarly, kaur et al. also reported that a compound named “Squalestatin analog 2” also interacted with residues including Arg45, Arg48, Tyr129, and Asn168, while Squalestatin analog 15 further interacted with Tyr248 (Kaur et al., 2019). Furthermore, Selvaraj et al. reported similar results in their study (Selvaraj et al., 2020). The conservation of the interaction of hit compounds with these residues across several studies suggest their potential pivotal role in the inhibition of this protein. Therefore, the hit compounds of this study can be considered viable potential inhibitors of this target. The interactions between CrtM and the hit compounds as well as the standard drug are further presented in Table 3.

**Table 3:** Interactions of the hit compounds and the standard drug (Methicillin) with the CrtM target.

		Interacting residues	
Compounds CID	Docking score (kcal/mol)	H-bonding	Hydrophobic interactions
136259988	−10.6	None	Phe22, Tyr41, Cys44, Arg45, Asp48, As49, Val111, Tyr129, Val133, Ala134, Val137, Gly138, Gly161, Leu164, Gln165, Asn168
44130717	−10.6	Tyr41, Arg45, Gln165, Tyr248	Ser19, Phe22, Phe26, Val133, Ala134, Val137, Gly138, Leu141, Leu145, Ala157, Leu160, Gly161, Leu164, Asn168, Ile241
44130601	−10.3	Tyr 41, Asn168, Tyr248	Ser19, Phe22, Phe26, Arg45, Val133, Val137, Leu141, Leu145, Ala157, Leu160, Ile164, Gln165, Phe233, Ile241
58628188	−10.3	Val133	Met15, Phe22, Phe26, Val37, Ile40, Tyr41, Arg45, Asp48, Ala134, Val137, Gly138, Leu141, Leu145, Ala157, Leu160, Gly161, Leu164, Gln165, Phe233
44130605	−10.2	Tyr41, Leu164, Gln165, Asn168	Ser19, Phe22, Phe26, Arg45, Val133, Val137, Gly138, Leu141, Leu145, Ala157, Leu160, Gly161, Leu164,
44130716	−10.1	Arg45, Tyr129, Gln165	Phe22, Phe26, Tyr41, Arg45, Asp48, Val111, Asp114, Val133, Val137, Leu164
Methicillin	−7.6	Asp48	Phe22, Phe26, Tyr41, Arg45, Ala134, Val137, Gly138, Ala157, Leu160, Gly161, Leu164, Gln165, Tur248

### 3.6. ADMET profiling of druglike hit compounds

The hit compounds that were evaluated to be druglike were further subjected to pharmacokinetics properties analysis and the results are presented in Table 4.

**Table 4:** The pharmacokinetics and toxicity profiles of the hit compounds.

Models	136259988	44130717	44130601	58628188	44130,716	Methicillin
Absorption and distribution						
HIA	100	97.36	57.57	90.58	57.96	45.34
Caco-2 permeability	0.636	0.969	1.073	1.243	1.074	0.563
P-gp (substrate)	Substrate	Substrate	Substrate	Non substrate	Substrate	Substrate
P-gp I (inhibitor)	Inhibitor	Inhibitor	Non inhibitor	Inhibitor	Non inhibitor	Non inhibitor
P-gp II (inhibitor)	Inhibitor	Inhibitor	Non inhibitor	Inhibitor	Non inhibitor	Non inhibitor
Distribution						
Blood–brain barrier	−0.968	−0.7	−0.625	−1.701	−0.621	−1.291
Central nervous system permeability	−1.902	−1.964	−3.142	−2.17	−3.911	−3.485
Fraction unbound	0.122	0.126	0.257	0.16	0.232	0.366
Metabolism						
CYP450 2C9 (inhibition)	Inhibitor	Non-inhibitor	Non-inhibitor	Inhibitor	Non-inhibitor	Non-inhibitor
CYP450 2D6 (substrate)	Non-substrate	Non-substrate	Non-substrate	Non-substrate	Non-substrate	Non-substrate
CYP450 2D6 (inhibition)	Non-inhibitor	Non-inhibitor	Non-inhibitor	Non-inhibitor	Non-inhibitor	Non-inhibitor
CYP450 3A4 (substrate)	Substrate	Substrate	Non-substrate	Substrate	Non-substrate	Non-substrate
CYP450 3A4 (inhibition)	Inhibitor	Non-inhibitor	Non-inhibitor	Inhibitor	Non-inhibitor	Non-inhibitor
CYP450 1A2 (inhibition)	Inhibitor	Non-inhibitor	Non-inhibitor	Inhibitor	Non-inhibitor	Non-inhibitor
CYP450 2C19 (inhibition)	Inhibitor	Non-inhibitor	Non-inhibitor	Non-inhibitor	Non-inhibitor	Non-inhibitor
Excretion						
Renal organic cation transporter 2 (OCT2)	Non-substrate	Substrate	Non-substrate	Non-substrate	Non-substrate	Non-substrate
Total Clearance	0.653	0.362	0.594	0.357	0.592	0.383
Toxicity						
Predicted LD50	2000 mg/Kg	2,600 mg/Kg	4,700 mg/Kg	1,000 mg/Kg	4,700 mg/Kg	2,880 mg/Kg
Predicted Toxicity class	4	5	5	4	5	5
Carcinogenicity	Active	Inactive	Inactive	Inactive	Inactive	Inactive
Mutagenicity	Active	Inactive	Active	Inactive	Active	Inactive
Cytotoxicity	Inactive	Inactive	Inactive	Inactive	Inactive	Inactive
Hepatotoxicity	Inactive	Inactive	Active	Active	Active	Inactive
Aryl hydrocarbon Receptor (AhR)	Inactive	Inactive	Inactive	Inactive	Inactive	Inactive
Androgen Receptor (AR)	Inactive	Inactive	Inactive	Inactive	Inactive	Inactive
Androgen Receptor Ligand Binding Domain (AR-LBD)	Inactive	Inactive	Inactive	Inactive	Inactive	Inactive
Aromatase	Inactive	Inactive	Inactive	Inactive	Inactive	Inactive
Estrogen Receptor Alpha (ER)	Inactive	Inactive	Inactive	Inactive	Inactive	Inactive
Estrogen Receptor Ligand Binding Domain (ER-LBD)	Inactive	Inactive	Active	Inactive	Inactive	Inactive
Peroxisome Proliferator Activated Receptor Gamma (PPAR-Gamma)	Inactive	Inactive	Inactive	Inactive	Inactive	Inactive
Nuclear factor (erythroid-derived 2)-like 2/antioxidant responsive element (nrf2/ARE)	Inactive	Inactive	Inactive	Inactive	Inactive	Inactive
Heat shock factor response element (HSE)	Inactive	Inactive	Inactive	Inactive	Inactive	Inactive
Mitochondrial Membrane Potential (MMP)	Inactive	Active	Inactive	Inactive	Inactive	Inactive
Phosphoprotein (Tumor Suppressor) p53	Inactive	Inactive	Inactive	Inactive	Inactive	Inactive
ATPase family AAA domain-containing protein 5 (ATAD5)	Inactive	Inactive	Inactive	Inactive	Inactive	Inactive

Notably, ADMET properties are meticulously evaluated at the early stages of drug discovery to assess their fitness for the later stages of drug development, particularly clinical trials. The assessment of the fitness is based on several parameters including the HIA, which portrays the ability of the drug to be taken up by the cells lining the small intestine and then transported into the bloodstream for distribution to their target organ, and ultimately the intended protein target. Interestingly, all the compounds including the standard drug (Methicillin) were predicted to possess good absorption, with the compounds with the CIDs “136259988,” “44130717,” and “58628188” predicting highly absorbable with maximum and near-maximum scores. This suggests that the compounds will possess high oral bioavailability if consumed by humans. Conversely, only one of the hit compounds with the CID “136259988,” was predicted to be non-Caco-2 permeable while the standard drug (Methicilin) was also predicted to be a non-permeant of the Cac0-2 cells. The P-gp is an important protein in the metabolism of drugs and other xenobiotics as it plays a role in the extrusion of substances outside of the cell. Assessment of the potential of the hit compounds of this study to serve as substrates or inhibitors of the protein revealed only the compound with the CID “58628188” as a non-substrate of the P-gp. Based on the results of this study, the hit compounds except for the compound with the CID “136259988” will likely require a high concentration to have the desired efficacy due to the effect of the P-gp action on the pharmacokinetics of the compounds. Similarly, the compounds with the CIDs “136259988,” “44130717,” “58628188” were all inhibitors of P-gp I and II. Consequently, the co-administration of the compounds along with other drugs could have restricted clinical usage due to potential drug–drug interactions and toxicity. Furthermore, the metabolism profiles of the compounds were assessed, and the assessment revealed some of the compounds will not serve as the substrate of the investigated phase I enzymes. Specifically, the compounds with the CIDs “44130601” and “44130716” were predicted to be non-metabolizable by CYP2D6 and CYP3A4 while the methicillin was also predicted to be a non-substrate of the enzymes. Also, the compound with the CID “136259988” was predicted to be an inhibitor of all the CYP isoforms that were investigated in this study, while other compounds except CID “58628188” were predicted to be non-inhibitors of the CYP isoforms. Precisely, CID “58628188” inhibit CYP2C9, CYP3A4, and CYP1A2. Noteworthy, the phase I metabolizing enzymes play a pivotal role in the metabolism of drugs, a process which renders them fit for their eventual elimination from the body. The ability of some of the compounds to serve as substrate of some of the enzymes alter the efficacy of the compounds, however, the exact effect needs to be further probed experimentally. Also, the inhibition of the enzymes by some of the drugs can contribute to drug–drug interactions via the inhibition of drugs that are metabolized by this enzyme.

The toxicity potentials of the compounds were further investigated. All the compounds were predicted to belong to toxicity classes 4 and 5, and their predicted LD50 was of high concentration. However, of all the hit compounds, only the compound with the CID “44130717” was found to be non-carcinogenic, non-mutagenic, non-cytotoxic, and non-hepatotoxic. Other compounds were found to possess toxicity potentials belonging to either one or more of the four categories. Administration of a mutagenic or carcinogenic compound as a drug could lead to the induction of deleterious mutations that could result in cancer development or aid its progression. Also, a cytotoxic and hepatotoxic compound has the potential to inflict damage to organs that are responsible for homeostasis, further leading to the development of diseases that may aid the progression of the one intended to be ameliorated by the drug. Therefore, only the compound with the CID “44130717” was selected for further downstream analysis, and it was considered the lead compound of the study.

### 3.7. FMO analysis and global reactivity descriptors calculation

The energies of the Frontier Molecular Orbitals (FMOs), specifically the Highest Occupied Molecular Orbital (HOMO) and the Lowest Unoccupied Molecular Orbital (LUMO) of the lead compounds, were determined based on the results obtained The energy values of the HOMO and LUMO for the drug-like hit compounds, as well as the standard drug (methicillin), are presented in Table 5. To gain further insights into the reactivity of the compounds, additional analysis was conducted by estimating various reactivity parameters. These parameters include Electron Affinity, Chemical Hardness (η), Chemical Softness (ζ), Electronegativity (χ), Ionization Potential, Electronic Chemical Potential (μ), and Electrophilicity Index (ω). These reactivity parameters were determined based on Koopman’s theorem (Hanwell et al., 2012).


(1) $$\text{Energy}\;\text{Gap}\;\Delta E=LUMO_{\varepsilon }-HOMO_{\varepsilon }$$

(2) $$\text{Ionization Potential}\;I=-E_{HOMO}$$

(3) $$\text{Electron affinity}\;A=-E_{LUMO}$$

(4) $$\text{Chemical hardness}\;\eta =\frac{1}{2}\left(\frac{\partial^{2}E}{\partial N^{2}}\right)V=\frac{1}{2}\left(\frac{\partial \mu }{\partial N}\right)V=\left(I-A\right)/2$$

(5) $$\text{Softness}\;\zeta =\frac{1}{\eta }$$

(6) $$\text{Electronegativity }\chi =-\mu =-\left(\frac{\partial E}{\partial N}\right)V=\left(I+A\right)/2$$

(7) $$\text{Chemical potential }\mu =\left(\frac{\partial E}{\partial N}\right)V=-\left(I+A\right)/2$$

(8) $$\text{Electrophilicity index}\;\omega =\frac{\mu 2}{2\eta }$$

**Table 5:** The computed values of the quantum mechanical properties of the hit compounds and the standard drug (Tucatinib).

S/N	Quantum chemical property	Compounds	
		44,130,717	Methicillin
1.	HOMO	−11.487 eV	−9.569 eV
2.	LUMO	−0.982 eV	−0.601 eV
3.	Energy gap (∆E)	10.505 eV	8.968 eV
4.	Ionization potential (I)	11.487 eV	9.569 eV
5.	Electron affinity (A)	0.982 eV	0.601 eV
6.	Chemical hardness (η)	5.252 eV	4.484 eV
7.	Chemical softness (ζ)	0.190 eV	0.223 (eV)^−1^
8.	Electronegativity (χ)	6.235 eV	5.085 eV
9.	Chemical potential (μ)	−6.235 eV	−5.085 eV
10.	Electrophilicity index (ω)	3.700 eV	2.883 eV

**Figure 4:**
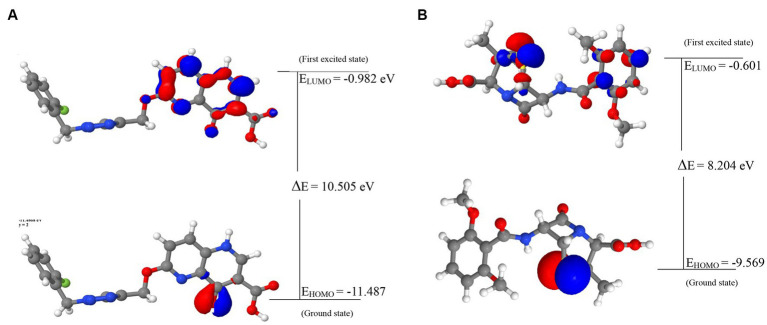
The frontier molecular orbitals of **(A)** 44130717. **(B)** Methicillin.

The visualization of the HOMO, LUMO, and band energies gap for the lead compounds and the standard drug (methicillin) were visualized. The values of these parameters offer critical insights into the reactivity and stability of the compounds, with the HOMO representing the highest energy level of the molecular orbitals that contain electrons. It represents the orbital with the highest energy where electrons are present in a stable molecule. Conversely, the LUMO is the lowest energy level of the molecular orbitals that does not contain any electrons. It represents an empty orbital in a stable molecule. Notably, the HOMO energy value of the lead compound was found to be highly negative relative to that of the standard drug (Methicillin), indicating a higher reactivity of methicillin. Conversely, the lead compound will be more stable compared to methicillin. The result of the chemical hardness and chemical softness further validates this inference (Figure 4).

### 3.8. Molecular dynamics study of the lead compound

#### 3.8.1. Structural stability and residual flexibility analysis

**Figure 5:**
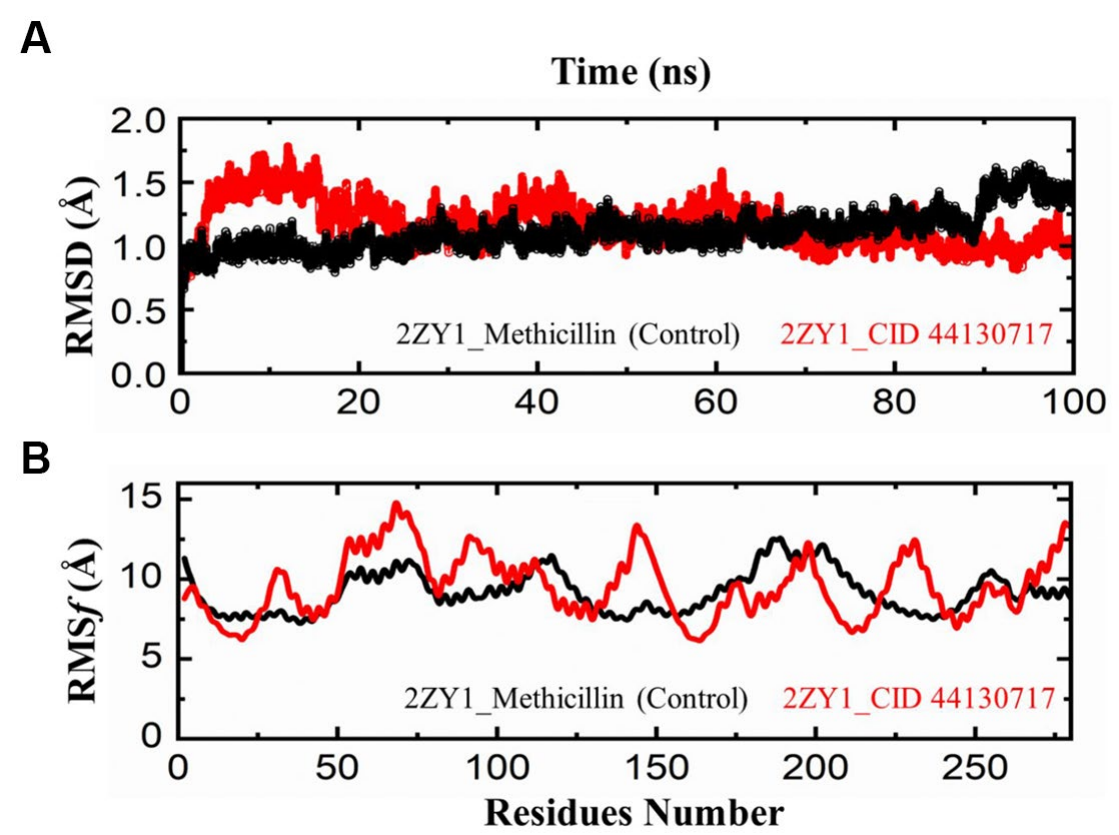
MD simulation trajectories analysis during the whole simulation period. **(A)** residual stability analysis of the 2ZY1_Methicillin (control) represented in Black, and 2ZY1_CID 44130717 hit represented in red colors. **(B)** residual flexibility analysis of the 2ZY1_Methicillin (control), and 2ZY1_CID 44130717 hit.

The comparative analysis of the Root Mean Square Deviation (RMSD) between the control structure (2ZY1_Methicillin) and the retrieved hit structure (2ZY1_CID 44130717) was conducted through a 100 ns molecular dynamics simulation using AMBER 22. The RMSD analysis aimed to assess the structural stability and conformational changes of the hit compound in comparison to the control. The RMSD comparison graph depicted in Figure 5A indicates notable differences between the control and hit structures. The control structure (2ZY1_Methicillin) exhibited a relatively stable RMSD between 0 and 85 ns simulation and after exhibiting an initial RMSD value of 1.5 Å. In contrast, the hit structure (2ZY1_CID 44130717) displayed a higher RMSD, from 0 and 25 ns simulation indicating greater deviations, and after the 25 ns simulation indicating stable RMSD in atomic positions during the simulation.

Moreover, The RMSF profiles of both the control and hit structures were calculated and examined. The resulting data were utilized to generate RMSF comparison graphs that illustrate the fluctuations of individual atoms across the protein sequences. Upon closer examination of the RMSF comparison graph depicted in Figure 5B, several distinct peaks are observed in both structures. These peaks denote regions of increased atom fluctuations, suggesting areas of higher flexibility or conformational variability within the protein structures. In the control structure (2ZY1_Methicillin), a prominent peak is evident in the alpha helix region, spanning residues 150 to 200. This peak indicates higher fluctuations in this region, potentially reflecting the natural flexibility of this portion of the protein. Conversely, in the hit structure (2ZY1_CID 44130717), two significant peaks are observed. The first peak occurs in region A (residues 50 to 100), indicating heightened fluctuations in this region. The second peak is evident in region B (residues 130 to 160), displaying increased fluctuations compared to the control structure.

#### 3.8.2. Structural compactness and hydrogen bond analysis

**Figure 6:**
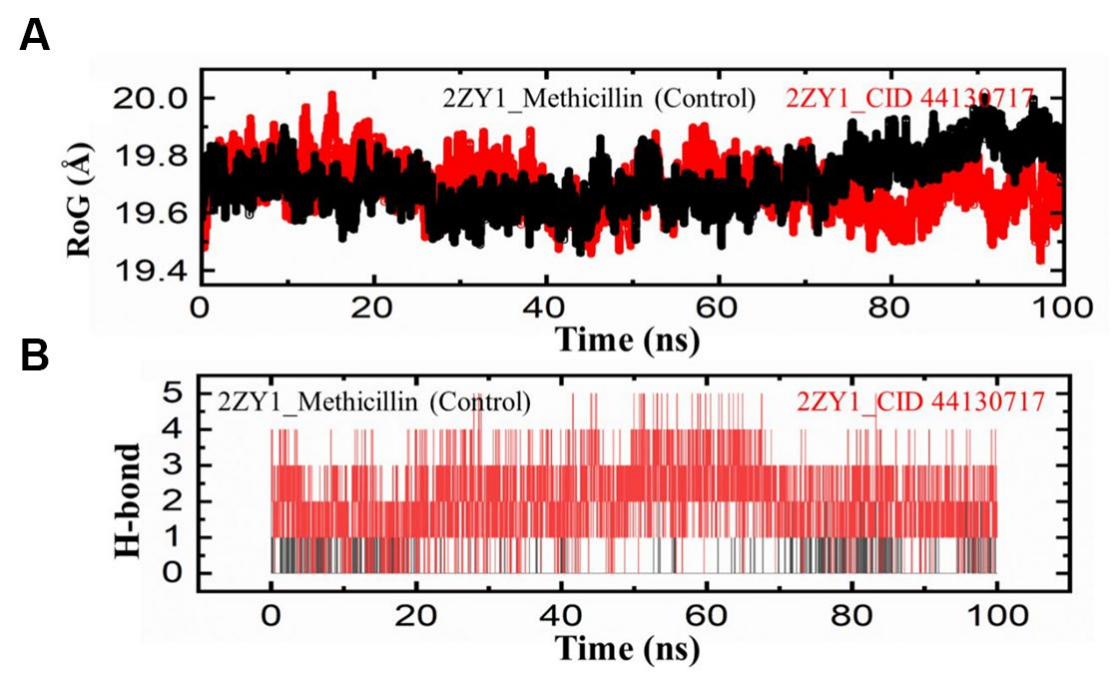
MD simulation trajectories analysis during the whole simulation period. **(A)** Structural compactness analysis of the 2ZY1_Methicillin (control), and 2ZY1_CID 44130717 hit. **(B)** Hydrogen bond analysis of the 2ZY1_Methicillin (control), and 2ZY1_CID 44130717 hit.

To further explore the structural characteristics of the protein structures, the Radius of Gyration (RoG) analysis was conducted and the results are depicted in Figure 6A. The RoG analysis provides insights into the compactness and overall size of the protein. The RoG values were calculated for both the control and hit structures throughout the simulation, and the resulting data were utilized to generate RoG comparison graphs. The RoG analysis offers complementary information to the RMSD and RMSF analyses, providing insights into the overall compactness and size variations of the protein structures. The RoG analysis revealed that the control structure (2ZY1_Methicillin) exhibited a relatively stable at the start and unstable at the end with an average RoG value of 19.8 Å, indicating consistent compactness and size throughout the simulation. In contrast, the hit structure (2ZY1_CID 44130717) showed an average RoG value of 19.5 Å, suggesting greater fluctuations at the start and stability at the end in compactness due to potential conformational changes induced by the hit compound. To investigate the potential impact of the hit compound on the protein’s interactions, a Hydrogen Bond (H-bond) analysis was conducted. The presence of hydrogen bonds between the protein and ligand can provide insights into stabilizing interactions and potential binding modes. The hydrogen bond analysis was performed throughout the simulation, and the number of hydrogen bonds formed between the protein and the hit compound was monitored. As evident in Figure 6B, during the simulation, the control structure (2ZY1_Methicillin) exhibited an average of 1 hydrogen bond. In contrast, the hit structure (2ZY1_CID 44130717) formed an average of 4 hydrogen bonds.

### 3.9. Binding free energy calculation

We conducted Molecular Mechanics/Generalized Born Surface Area (MM/GBSA) analysis to evaluate the binding free energy and enhance our comprehension of protein-ligand interactions. The MM/GBSA computations were executed for the protein-ligand complexes that were sampled during the molecular dynamics simulations. The binding free energy (ΔG_bind) is expressed as the sum of various factors, including the energy from molecular mechanics (ΔG_MM), solvation energy (ΔG_GBSA), and entropy contribution (ΔS). Additionally, the standard deviation (SD) of the computed values is provided to indicate result reliability. The calculated ΔG_bind values indicate the overall binding affinity of the ligands to the protein. Negative ΔG_bind values suggest favorable binding, while positive values indicate unfavorable binding. In our study, 2ZY1_Methicillin (control), and 2ZY1_CID 44130717, displayed ΔG_bind values of-33.4996 ± 2.1221Kcal/mol, −46.057 ± 4.2763 kcal/mol, respectively, indicating strong binding affinities. Overall, the MM/GBSA analysis provided valuable insights into the binding free energy and the key molecular interactions between the protein and ligands. These findings enhance our understanding of the structure–activity relationships and guide future drug design efforts targeting the protein of interest.

## 4. Conclusion

In this study, we employed CADD techniques to probe the potentials of napthyridine derivates to serve as potential inhibitors of CrtM of *S. aureus*. Notably, of all the sixty-four compounds that were subjected to comprehensive molecular modeling techniques including molecular docking, Ro5 screening, pharmacokinetics properties prediction, quantum mechanical calculations, and molecular dynamics simulation. Only a compound named 6-[[1-[(2-fluorophenyl)methyl]triazol-4-yl]methoxy]-4-oxo-1H-1,5-naphthyridine-3-carboxylic acid was found to possess suitable drug-like potentials and void of any potential toxicity issues.

## Data availability statement

The raw data supporting the conclusions of this article will be made available by the authors, without undue reservation.

## Author contributions

MB: Investigation, Writing – original draft. MS: Investigation, Investigation, Writing – original draft. GZ: Software, Writing – review & editing. YB: Methodology, Writing – original draft. BS: Formal Analysis, Methodology, Writing – review & editing. LO: Investigation, Methodology, Writing – review & editing. FK: Investigation, Methodology, Writing – original draft, Writing – review & editing.

## Footnotes

^1^ https://pubchem.ncbi.nlm.nih.gov/

^2^ https://www.rcsb.org/

^3^ https://www.swissadme.ch.index.php/

^4^ https://biosig.lab.uq.edu.au/pkcsm/prediction

^5^ https://tox-new.charite.de/protox_II/
